# Assessing the Value of Integrated Evidence Approaches in Drug Development

**DOI:** 10.1007/s43441-025-00778-y

**Published:** 2025-04-23

**Authors:** Joseph A. DiMasi, Melvin Skip Olson, Zachary Smith, Kenneth A. Getz, Gorana Capkun

**Affiliations:** 1 Tufts Center for the Study of Drug Development, Tufts University, 145 Harrison Avenue, Boston, MA 02111 USA; 2 Olson Strategies GmbH, Allschwil, Switzerland; 3 Merck Healthcare KGaA, Darmstadt, Germany

**Keywords:** Integrated evidence, Clinical development phases, Expected net present value, Strategic decision making, Value of evidence

## Abstract

**Background:**

The use of Integrated Evidence Plans (IEPs) by the pharmaceutical industry has expanded in recent years with the aim of optimizing healthcare and patient outcomes. The evidence base of IEPs goes beyond traditional randomized controlled trials to provide holistic evidence suitable for all stakeholders and allows for consideration of different packages in different regions. However, this approach to drug development is not systematically adopted by all sponsors because of perceived uncertainty in its investment value.

**Methods:**

We introduce the concept of value drivers to which we apply an expected net present value (eNPV) model of the cash flows for drug development and commercialization. The approach is outlined for two, typical, hypothetical lifecycle management IEPs. The measure of IEP value is defined as the increment in eNPV that occurs when IE programs are employed in comparison to when they are not.

**Results:**

We found substantial value for IEPs. One example incorporated a plan to conduct an observational study that could be used as a basis for approval in lieu of a classical phase II trial for a supplemental indication. In the other example, increased adoption of the new treatment leads to a highly positive increment in eNPV based on the critical evidence generated in a phase IIIb study.

**Conclusions:**

Use of value drivers and eNPV-based value models when planning for IEPs can provide objective guidance for project teams. The value can be estimated through formal economic analysis that considers planned timelines, R&D costs, estimates of the likelihood of regulatory approval, patient access and clinical adoption if development is successful.

**Supplementary Information:**

The online version contains supplementary material available at 10.1007/s43441-025-00778-y.

## Introduction

There has been a growing trend in the pharmaceutical industry to concentrate on the concept of Integrated Evidence (IE). The former approach of many compartmentalized and mainly sequentially applied approaches to evidence generation has led to conflicting priorities and unclear decision making that has ultimately not always been in the best interests of patients and other stakeholders. Integrated Evidence includes phase 1–4 clinical trials, PK/PD trials, and real-world evidence (RWE) studies as well as activities designed to investigate disease and clinical practice.

Integrated Evidence Plans (IEPs) are strategic tools to comprehensively plan evidence generating activities across various functions over a product’s lifecycle to meet the diverse needs of healthcare stakeholders. They serve many key purposes including a holistic approach to evidence, cross-functional collaboration, long-term planning, stakeholder alignment, resource optimization, and regulatory and market access support. The types of evidence considered are varied and meant to be all-encompassing. Examples would be clinical trials, modeling studies, RWE studies, literature reviews and evidence synthesis, and network meta analyses [1, 2]. 

Between 2011 and 2021, there was a ten-fold increase in the number of new Food and Drug Administration (FDA) approvals based, in part, on data and evidence gathered in clinical care, community and home-based settings (e.g., natural history studies; patient and electronic health records; claims data and registries) [3]. A separate study forecasts a nearly 15% annual growth rate in the real-world data (RWD) market between 2022 and 2026 [4]. 

There are many factors driving this promising trend in the use of IE for decision-making for pharmaceutical products. Among health care and clinical research stakeholders, for example, there has been increasing recognition of the need to efficiently translate scientific evidence on investigational treatments gathered in controlled clinical trial settings into optimal use within clinical care settings [5]. An extensive body of research in the literature consistently demonstrates a highly inefficient translational gap between clinical research and clinical care [6, 7]. The translational gap is also the subject of the growing field of implementation science that relies on evidence using qualitative methods, hybrid clinical trials, and RWD [8]. The use of RWD in IEPs in particular offers the advantage of including data on patients who are not typically included in RCTs.

Patient communities and advocacy groups, particularly in rare diseases, have echoed the important role that RWE can play in reducing translation delays with appropriate consent and privacy protection and therefore enable faster access to medicines [9]. Many believe that using all available evidence – scientific, observational and personal – will enhance public and patient trust and engagement and will more rapidly identify and provide the most clinically-meaningful and relevant outcomes as safely as possible [9]. Indeed, this leads to the concept of integrated evidence (IE) that combines evidence from all possible sources, including RCTs, into one integrated evidence plan (IEP) that seeks to optimize an evidence package that will satisfy the needs of all stakeholders simultaneously.

An IEP is used to identify evidence needs for the relevant stakeholders (internal and external). At early stages in the drug development process, the IEP focuses on shorter term goals like proof of concept, mechanism of action, the scientific story, epidemiology, unmet needs, and the performance of the competition (or standard of care) while also optimizing success in planning and execution of clinical trials. At later phases, IEP can be seen as an enabler of external decision-making. As drug development proceeds, the IEP is updated by incorporating new information and adapting to new challenges.

At the same time, in an ongoing effort to bring innovation to patients in an efficient way, pharmaceutical and biotechnology companies are looking to optimize clinical trial performance. A recent study, for example, reported a tripling in the number of procedures performed and a seven-fold increase in the volume of data collected per pivotal trial during the past 15 years. In that same period, clinical trial durations, participation drop-out rates, and the mean number of protocol amendments increased significantly [10]. The use of RWE holds promise in helping to optimize clinical trial planning, performance and cost through early identification of target populations and endpoints/outcomes; better investigative site selection and targeted recruitments [11]. 

The unprecedented growth in applications and technologies that capture and more easily access patient health information has improved the ability to integrate data across multiple clinical research and clinical care systems. Ultimately this cross-platform interoperability and integration will better inform and monitor more comprehensive and flexible patient care [12]. The Food and Drug Administration (FDA) and the European Medicines Agency (EMA) recognize the important role that these data management solutions can play and they have been encouraging the use of IE to gather more complete evidence demonstrating safety and efficacy, accelerate approvals and to enable a continuous learning health system [12]. 

Despite growing recognition and use of RWE, the adoption of integrated evidence in drug and biologic registration programs during the past decade has been muted. Pharmaceutical and biotechnology companies – organizations that fund the vast majority of clinical trials of new medical therapies – face several barriers [13]. It has been difficult, for example, to coordinate evidence generation activities as it is a shared responsibility among a fragmented collective of functional areas including research, clinical development, clinical operations, medical affairs, data management and health economics and outcomes [14]. Sponsor companies have expressed concerns about the level of investment required to fully support the generation of integrated evidence and the value proposition in doing so [15, 16]. 

To assist research sponsors in evaluating the return on investment, we developed a framework to assess the value of evidence, with the goal being to allow the comparison of different IEPs to enable data-driven decision making in terms of evidence needed to better meet patient needs.

This paper presents a novel value framework to develop a patient-relevant IEP along with relevant expected net present value (eNPV) calculations to quantify its value. An eNPV analysis is a commonly used, and widely accepted, risk-adjusted financial modeling technique for industrial investments. To our knowledge, the systematic use of a value framework combined with a quantitative component has not been performed previously. It is our hope that this analytical structure will inform decisions regarding commitment to and investment in the use of RWE and IE as they have the potential to have a significant impact on improving patient outcomes.

## Data and Methods

A literature search was conducted to determine what, if any, benchmarks had been established for drug development programs that used an IE model. Literature searches were conducted for the terms “Integrated Evidence” and “Learning Health Model.” The search resulted in only a few publications which were mainly theoretical and included little quantitative evidence. Additional literature searches were conducted for publications regarding payer attitudes and perceptions about the use of real-world evidence, and the impact of real-world evidence on product launches. These searches resulted in additional publications being identified but did not provide useful benchmarks or frameworks that could be incorporated into a quantitative model such as eNPV [11–14, 17−23].

Therefore, in absence of established benchmarks for drug development programs using an IE model, the hypothesized data used for model parametrization were based on two illustrative examples chosen to represent realistic challenges faced by the pharmaceutical industry. In one example, the additional element in the IEP consisted of an RWE study and in the other, a phase IIIb trial.

### IEP Value Framework

The IEP value framework was developed as a way to portray and concretely express the value that an IEP brings such that one IEP can be compared to another. The value drivers mainly consider the benefit that the IEP brings to the approval and use of an asset in the marketplace. The Time of Availability defines the time at which the product is first available to patients in a given market. Naturally, an earlier Time of Availability means that patients benefit by having an earlier access to the product. The Probability of Success is the probability of achieving patient availability and includes the probability of regulatory success and/or the probability of achieving reimbursement/access. A higher Probability of Success increases all valuations made in the quantitative assessment as they take time and risk into account. The Adoption of a product measures the speed of uptake of a new product in a given healthcare system and might benefit, for example, by having evidence that makes it clear to prescribing physicians which patients are mostly likely to benefit from the product. The Number of Patients Reached is a measure of the number of patients receiving the new therapy. The End of Exclusivity is the time at which a therapy loses marketing exclusivity. As all IEPs have an inherent cost associated with their execution, Cost is considered as one of the value drivers. The value driver definitions are shown in Table 1. It is worth noting that these are broad categories and they can be split into subcategories to highlight individual effects, which would be meaningful depending on the application.

**Table 1:** Value driver definitions

Value Driver	Definition
Time of Availability	Time during which the product is available for patients in a given market.
Probability of Success	Probability of achieving patient availability. This includes the probability of regulatory success and/or the probability of achieving reimbursement/access.
Adoption	Speed of uptake of a new therapy in a given healthcare system.
Number of Patients Reached	Number of patients receiving a new therapy.
End of Exclusivity	Time at which therapy loses marketing exclusivity.
Costs	Costs associated with the incremental IEP component(s) including studies, training and educational programs, etc.

The value drivers, as presented in Table 1, are broad in nature. There are many facets of value that can be elicited for each value driver. For example, Time of Availability hinges on both the time of regulatory approval and the time of initial reimbursement. Notably, in the United States and Germany, these dates are essentially the same, but most other countries go through a separate process to assess if and how reimbursement will be granted. Evidence activities can be done to shorten the duration of the phase 3 program that would lead to an earlier regulatory approval and/or evidence activities can target the reimbursement process in an attempt to shorten that process. Hence, both Regulatory and Reimbursement are subcategories of the broad value driver, Time of Availability. Similarly, Physician, Healthcare System, Patient, Medical Societies, and Guidelines could all be subcategories of the value driver Adoption.

Each incremental component, or group of components, of an IEP is assessed against its impact on each of the value drivers. The fundamental idea behind the value framework is that if a proposed IEP does not have a tangible impact on any of the value drivers, then it should not be proposed as it is not bringing tangible value to the patients or sponsor.

One of the case studies considers an already-approved asset being developed for a supplemental rare disease indication. The proposed IE option involves adding an observational study as a basis for regulatory approval in one region in combination with a pharmacokinetic (PK)/bioequivalence study. To support regulatory decision making in other regions, a phase II trial with a later read-out would also be part of the IEP plan.

The second case study involves an already-approved asset, where the proposed IEP includes the additional component of conducting a phase IIIb randomized clinical study to support a new indication. We consider, for illustrative purposes, both the base plan, in which the results of the phase IIIb study are available two years after Time of Availability for the new indication and a more aggressive integration of the plan whereby the same study reads out at the Time of Availability.

## Expected Net Present Value Model

To quantify the value of adopting IEPs we employed a methodology that has been widely accepted and utilized across industries for evaluating the value of investment projects. The eNPV method accounts for R&D investment cash flows, risks in reaching the marketplace, costs of commercialization, and the projected number of patients reached. To account for the cost of delay between investments and returns, cash flows from different periods are made comparable through discounting. For industrial investment projects the discount rate to be applied to the cash flows is a company-specific cost of capital.

The critical data needed for eNPV analysis for drugs in development include the duration of clinical trials and non-clinical studies, the likelihood of proceeding from one development phase to the next phase, R&D costs for development phases, and annual projected sales. We can measure the value of change in any or all of these factors with an eNPV model. For our purposes, the eNPV of a given IEP can be compared to that from another IEP to provide additional input to the decision-making process to help with the rational allocation of investment capital to patient outcomes derived.

This general method has been applied recently to a number of hypothesized improvements to the drug development process, including adopting patient engagement methods [24], integrated formulation development, real-time manufacturing and clinical testing [25], single-source versus multi-vendor outsourced biopharmaceutical manufacturing [26], and decentralized clinical trial methods [27]. What we model here are incorporation of indicative IEPs into drug development lifecycle management programs for two different case studies. The basics of value drivers and the eNPV valuation method are illustrated by these two examples.

Aside from utilizing internally generated assumptions about the timing of development and regulatory review, the cost of development, and forecasted future sales if development is successful, we need to choose values for certain financial parameters that can be applied to all projects. However, we also provide sensitivity analysis results around these parameter choices as well as the main assumptions for the eNPV calculations in an online Supplementary Data File. The modeling also makes it possible to conduct sensitivity analysis around any of the forecasted timing, costs, and sales parameters.

Aside from assessing the value of IE plans as increments in eNPV, the results can also be presented in the form of a return on investment (ROI) metric as shown in the Supplemental Data File. In each case, the ROI is defined as the eNPV delta from implementing the IE plan divided by the expected net present value of the costs of any additional activities engendered by the IE plan.

## Results

To illustrate how value drivers, in combination with an eNPV model, may be generally used to develop, test, and value the myriad types of IEPs, we conduct full analyses for two different IEPs. As noted, certain financial parameters are applied to projects across the board. Sensitivity analyses around these values are reported in tables in the Supplemental Data File.

### Case Study 1: Lifecycle Management of Drug Asset (New Indication)

The first IE case we consider involves an already-approved drug being investigated in a new, rare disease indication without a clearly defined standard of care. We assume that there are regional differences in regulatory requirements for approval in this supplemental indication whereby one region will accept an observational study combined with a PK/bioequivalence study and the remaining regions will require a PK/bioequivalence study and a phase II clinical trial and put little weight on an observational study. The strategic question of interest then becomes, given the PK/bioequivalence study and the phase II clinical trial will be conducted regardless of strategic assessments, does the additional investment in the observational study for the one region benefit the program and patients overall?

Consider first the value drivers impacted by the strategic question. A PK/bioequivalence study and phase II trial will be conducted to serve all regions, and one region will accept an observational study in place of the phase II trial. Since the phase II trial will be conducted in any case, it seems that the observational study is simply an extra cost and does not appear to be a wise investment. However, the duration of both the PK/bioequivalence and observational studies would typically be just under 1 year whereas a phase II clinical trial would typically run for just under 3 years. The regulatory submission in the one region could be made two years earlier if the observational study is conducted and, hence, have a major impact on the Time of Availability value driver. As patients in that region would have availability earlier, patients would be reached already at the earlier date. In addition, we assumed that the Probability of Success value driver would increase by 10% for the regions that will not formally include the observational in their approval package due to additional evidence and an increased number of patients exposed to a new treatment. No impact on the other value drivers is foreseen in this case study.

**Fig. 1:**
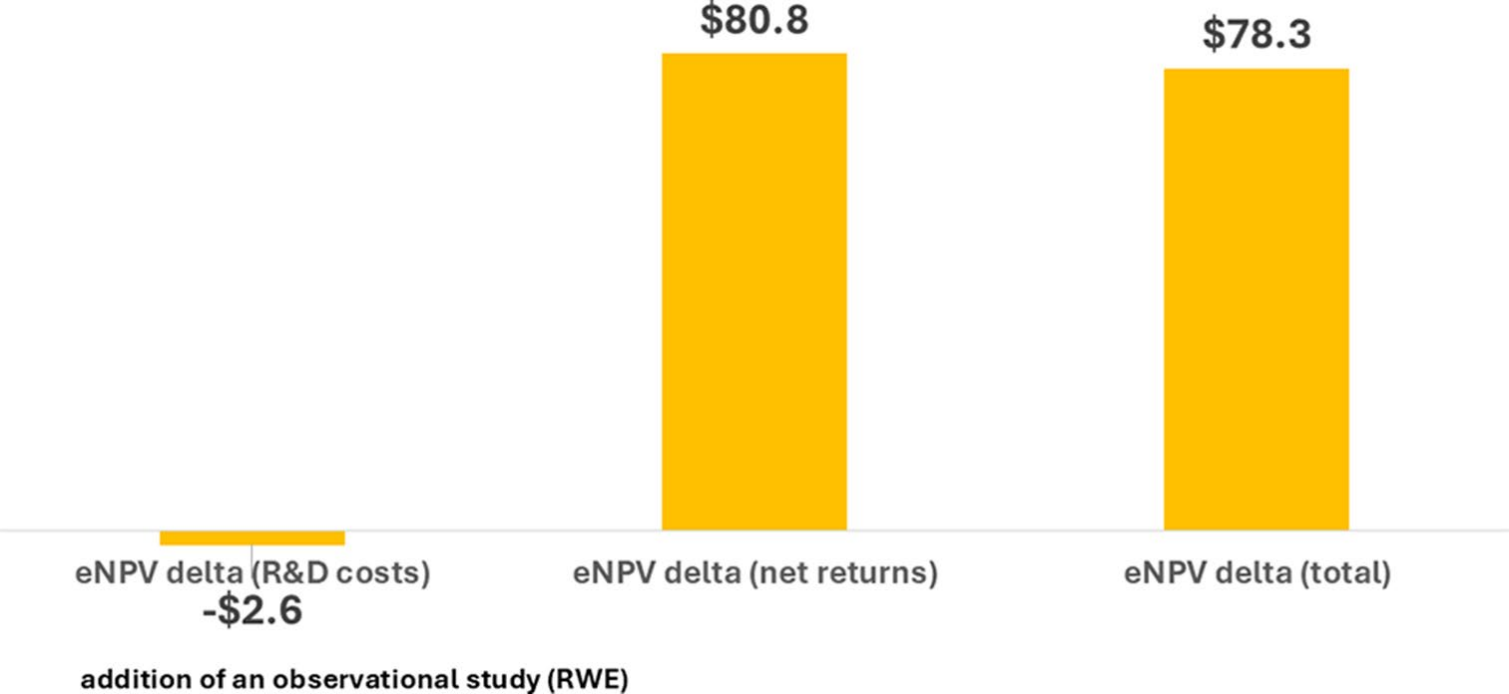
Change in eNPV with an IEP (all regions) for Lifecycle Management (new rare disease indication) of a Drug Asset (millions 2020 USD)

With an assessment of the impact on value drivers complete, the eNPV model was used to quantify a projected benefit to patients. In Fig. 1, the eNPV is shown considering development costs only, net returns only, and combined. The change in eNPV considering just R&D costs is negative (-$2.5 million) because an additional study is conducted. The increment in eNPV for net returns is substantially positive ($80.8 million) because the shorter Time of Availability in one region and slightly higher Probability of Success in the others translate to more patients receiving medication at an earlier time point. The change in total eNPV of $78.3 million represents the net benefit of making an extra investment to increase the projected number of patients reached earlier. The positive eNPV delta is robust to changes in discount rate, contribution margin, and probability of success (Supplemental Table S1).

### Case Study 2: Lifecycle Management of an Asset (Support New Indication)

The second case study involves an effort to demonstrate the effective use of early treatment for the original regulatory approval population in a specific region. In this case, the IEP involves the addition of a phase IIIb trial. The value drivers impacted by such a study are those of Adoption and Number of Patients Reached. The results should support better and quicker diagnoses of patients as well as earlier inclusion of the asset in the treatment options for the disease. In turn, this will both quicken and strengthen adoption of the product and lead to both more patients reached and better lifelong outcomes for patients.

The rationale for the notion that diagnostic tools that can be used in RCTs can also be accessible in clinical practice can be outlined as follows. The design of the phase IIIb trial we are considering comprises two phases: active vs. placebo in the first phase, and active (a) vs. active (b) in the second phase, whereby the (a) group has received the active for the entire study and the (b) group has received placebo followed by active. The comparisons at the end of the first phase and at the end of the second phase should support the claim, if significant, that early diagnosis and early treatment of the disease are important for the long-term well-being of patients. The results will reinforce the importance of early screening, use of diagnostic tools, protocols and guidelines to enable the earlier diagnosis and use of the treatment early in the patient journey.

**Fig. 2:**
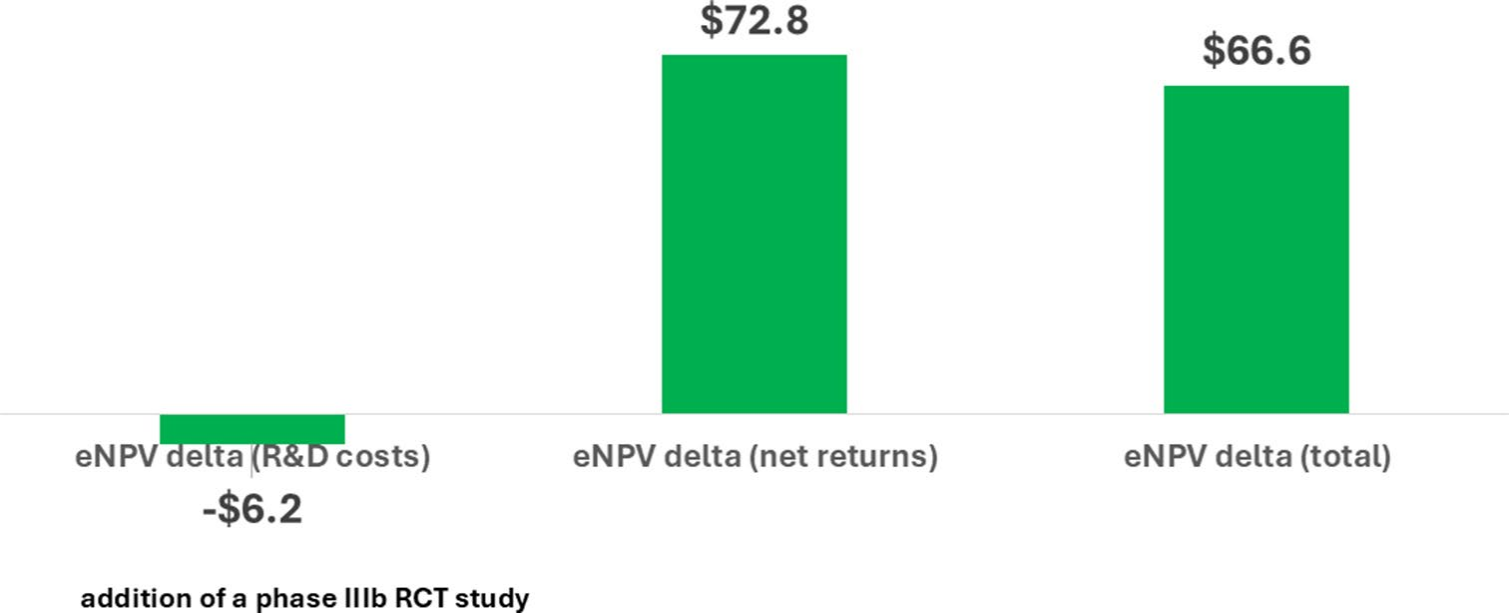
Change in eNPV with an IEP (all regions) for Lifecycle Management (expanded patient population) of a Biologic Asset (millions 2020 USD)

Figure 2 shows that the reduction in eNPV due to the extra cost for the trial is $6.2 million. However, the improvement in Adoption more than counterbalances the costs and results in a benefit of $72.8 million. Thus, on net, the increment in eNPV for the IEP is $66.6 million. The results are not particularly sensitive to wide variations in the assumed cost of capital or the contribution margin (Supplemental Table S2).

**Fig. 3:**
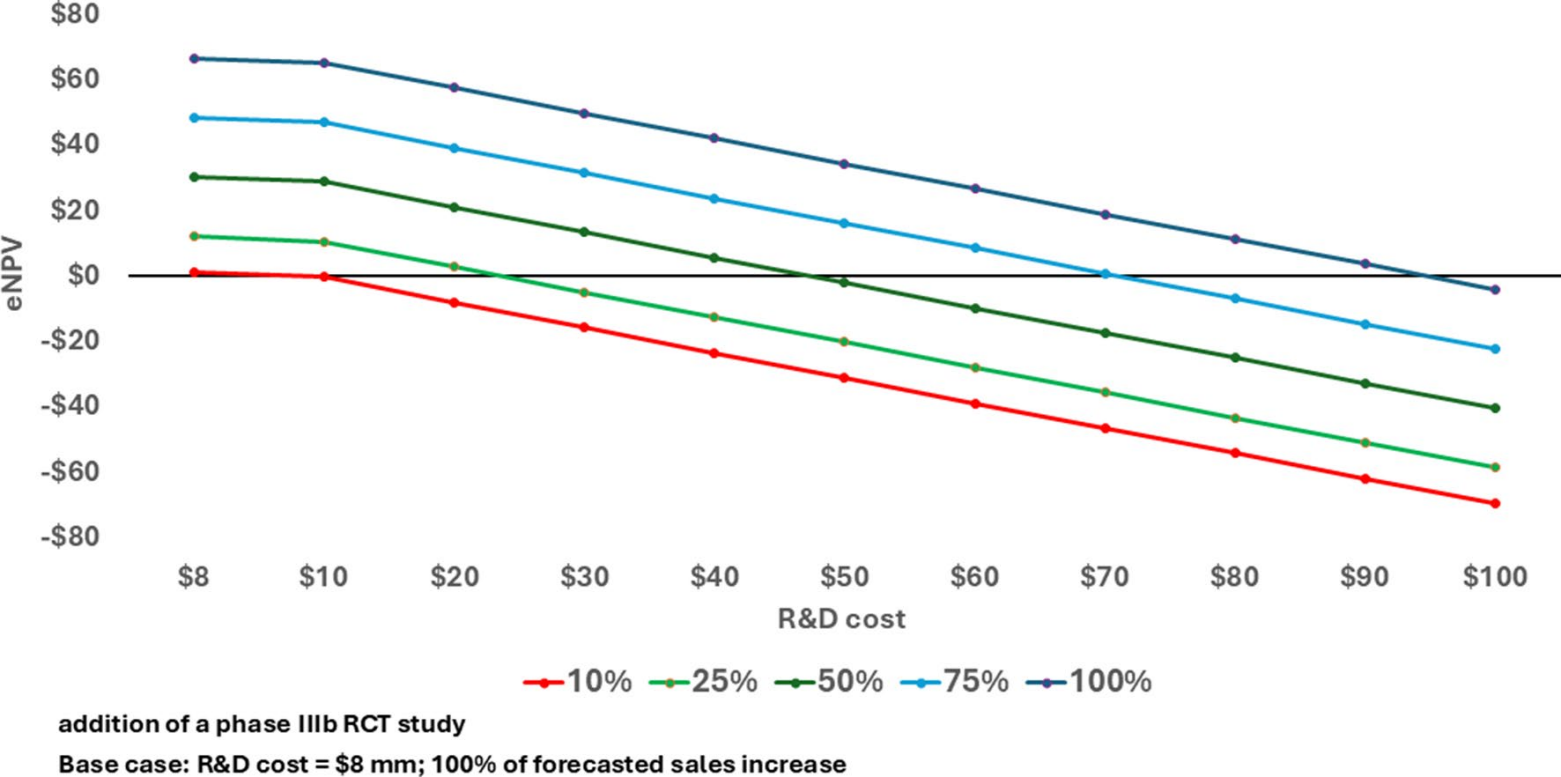
eNPV at various phase IIIb development costs and percent of forecasted sales increase with an IEP for lifecycle management (expanded patient population) of a biologic asset (millions 2020 USD)

Key assumptions for this case study are the forecasted impact on Adoption and the anticipated increase in Number of Patients Reached. The sensitivity of the results to those assumptions is illustrated in Fig. 3. If the cost of the phase IIIb study is $8.0 million in nominal dollars, the IEP yields a positive change in eNPV even if the Adoption only has 10% of its projected impact. If the assumed impact of Adoption is accurate (100% in the chart), the cost of the phase IIIb trial would have to be more than 11 times higher than estimated before the change in eNPV is negative. The positive results for this IEP are therefore highly robust to different assumptions about parameter values (Supplemental Table S3).

Integration of appropriate evidence earlier in a product life cycle is thought to be crucial for optimizing drug development and commercialization [11]. Consequently, we also examine the impact of introducing the IEP earlier in the drug development process. Specifically, instead of beginning the phase IIIb trial at the Time of Availability, we instead assume that it is started two years earlier and has a duration of two years.

**Fig. 4:**
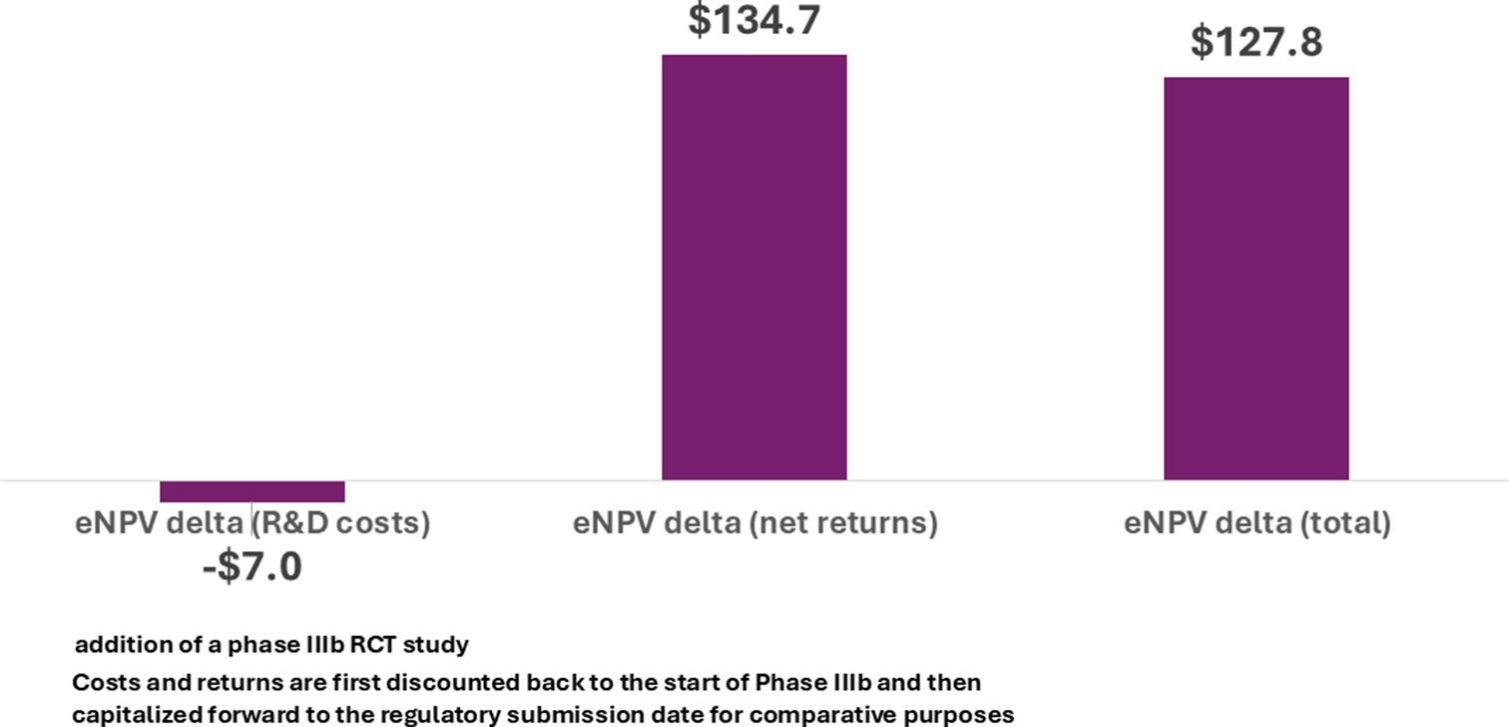
Change in eNPV with an IEP for lifecycle management (expanded patient population) of a biologic asset completed two years earlier (millions 2020 USD)

Figure 4 shows the top line results. The overall increment in eNPV ($127.8 million) is nearly twice as high as what we found for the scenario where the trial is initiated upon original submission for marketing approval of the investigational drug ($66.6 million). As before, the results are robust with respect to substantial variation in assumptions.

**Fig. 5:**
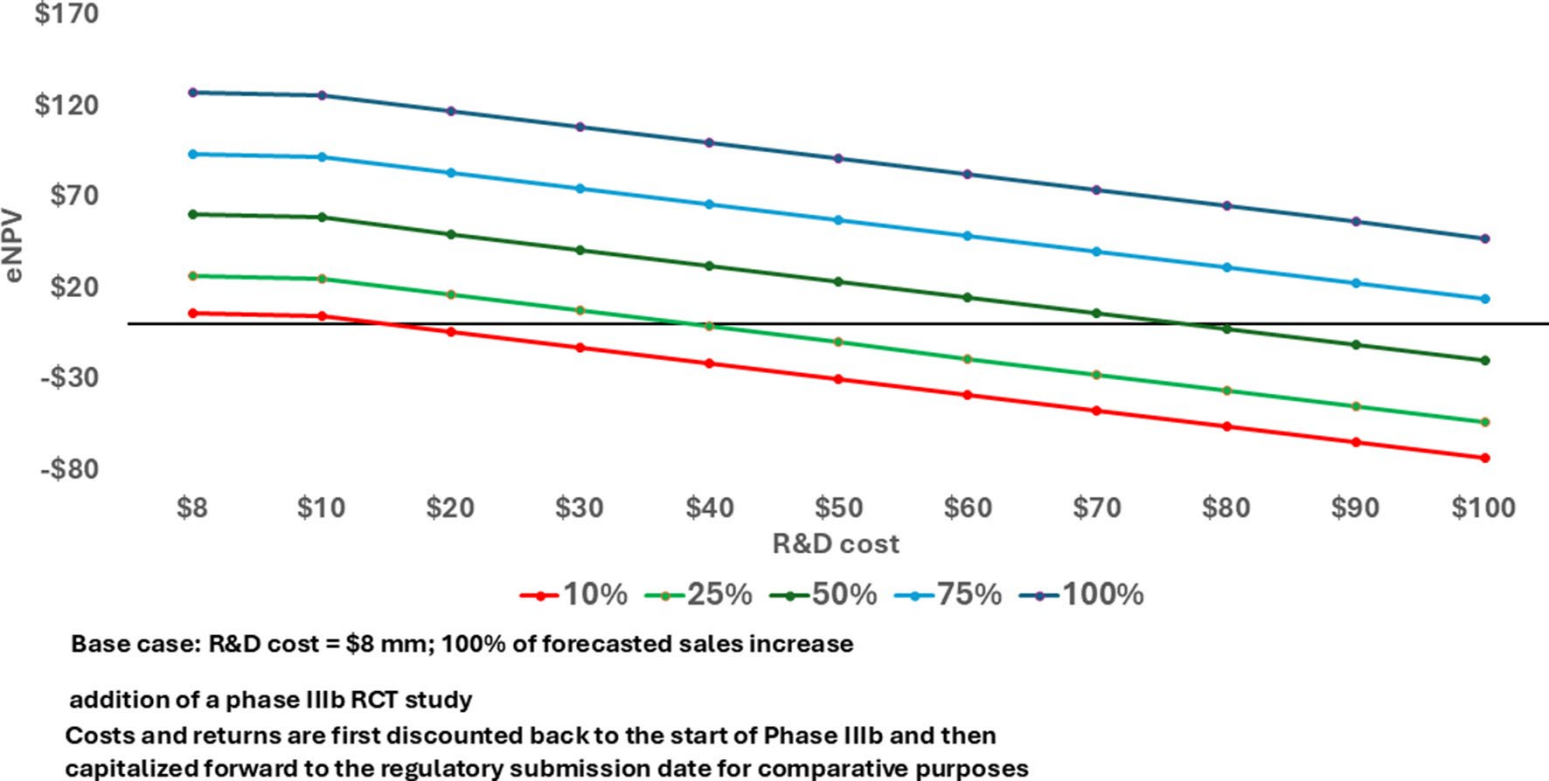
eNPV at various phase IIb development costs and percent of forecasted sales increase with an IEP for lifecycle management (expanded patient population) of a biologic asset completed two years earlier (millions 2020 USD)

In Fig. 5 we examine the relationship between trial cost and the increment in eNPV from changes in the Adoption and Number of Patients Reached value levers and we see that the results are more strongly positive for implementing the IEP two years earlier in the product lifecycle. The very strong value of the evidence coming from such a trial performed earlier in the product lifecycle breaks with the typical sequential form of decision making while controlling for risks in the eNPV model.

## Conclusions

Integrated evidence generation by pharmaceutical firms has expanded substantially in the last decade, but challenges to widespread adoption remain [10, 13, 22]. The ways in which IE can be integrated into the drug asset development and commercialization lifecycle are numerous, complex, and highly varied. Firms have historically routinely done financial analysis for investment in investigational drugs under a traditional development and commercialization paradigm. This has frequently been accomplished with eNPV analysis. Decisions about introducing IE activities can also benefit from expanding formal economic analysis to IEPs. It will require careful consideration of how the plans can impact the value drivers throughout the product lifecycle and realistically estimating those impacts.

The results of our analyses for two very different IEPs were robust and would have enabled teams to optimize evidence generation packages by choosing the one with the largest gain in overall value, if budgets restricted implementation to just one. A plan to conduct an observational study in lieu of a phase II trial for a new indication of a drug asset in one region, with value being driven through earlier Time of Availability and an increased Probability of Success, was associated with an important increase in eNPV. The second plan involved conducting a randomized phase IIIb study that could encourage more optimal Adoption and an increase in the Number of Patients Reached that translate into a highly positive increase in eNPV. Initiating the plan (the phase IIIb study) two years earlier in the process yielded a nearly doubling of the eNPV.

We have illustrated here how the strategic use of value drivers can be supplemented with a formal financial analysis using an eNPV platform. The eNPV is a financial metric that is already widely used in the pharmaceutical industry as a tool to assist in objective decision making. That approach is highly flexible and can account for details about numerous development opportunities simultaneously as well as consideration of its adoption in a single country, a single region or globally. It is also highly amenable to detailed sensitivity analysis around parameters whose values involve some uncertainty.

Although assessing the impact of the value drivers is inherently difficult, if enough evidence accumulates over time, it is conceivable that variation in parameter values can be accommodated by Monte Carlo simulations for the eNPV platform. This would further increase confidence in the method and results. We hope that this approach can further motivate pharmaceutical companies to regularly consider IEPs to create decision-ready evidence for multi-stakeholder engagement offering them an objective framework for assessing the impact of their evidence activities early in the drug development process.

## Electronic Supplementary Material

Below is the link to the electronic supplementary material.

Supplementary Material 1

## Data Availability

No datasets were generated or analysed during the current study.

## References

[1] Mckinsey. & Co. Integrated evidence generation: a paradigm shift in biopharma. December 3, 2023, https://www.mckinsey.com/industries/life-sciences/our-insights/integrated-evidence-generation-a-paradigm-shift-in-biopharma. Accessed February 21, 2025.

[2] Grueger J, Andre N, Vaidyanathan S. The future of integration evidence planning in biopharma. BCG, April 27, 2023, https://www.bcg.com/publications/2023/the-future-of-integrated-evidence-planning-in-biopharma. Accessed February 21, 2025.

[3] Aitkin M, Kleinrock M, Connelly N, et al. Global trends in R&D: 2021 overview. IQVI Institute Report; February 2022. p. 11.

[4] MarketsandMarkets. (2022). Real world evidence/RWE solutions market by component - global forecast to 2026. https://www.marketsandmarkets.com/Market-Reports/real-world-evidence-solution-market-76173991.html. Accessed Jan 20, 2023.

[5] Booth C, Tannock I. Randomized controlled trials and population-based observational research: partners in the evolution of medical evidence. Br J Cancer. 2014;110:551–5. 24495873 10.1038/bjc.2013.725 PMC3915111

[6] Lenfant C. Clinical research to clinical practice– lost in translation? N Engl J Med. 2003;349:868–74. 12944573 10.1056/NEJMsa035507

[7] Abu-Odah H, Said N, Nair S, et al. Identifying barriers and facilitators of translating research evidence into clinical practice: A systematic review of reviews. Health Social Care. 2022;30(6):3265–76. 10.1111/hsc.13898 35775332

[8] Olson M, Rootkin L. The triple win– implementation science benefits patients, healthcare systems and industry alike. JCER. 2022;11(9):639–42. 10.2217/cer-2022-0058. 35481349 10.2217/cer-2022-0058

[9] Oehrlein E, Graff J, Harris J, et al. Patient-community perspectives on real-world evidence: enhancing engagement, Understanding and trust. PCOR. 2019;12:375–81. 10.1007/s40271-019-00356-z PMC6598955 30666526

[10] Getz K, Smith Z, Kravet M. Protocol design and performance benchmarks by phase and by oncology and rare disease subgroups. Therapeutic Innov Regul Sci. 2023;57(1):49–56. 10.1007/s43441-022-00438-5 PMC9373886 35960455

[11] Olson M. Can real-world evidence save pharma US$1 billion per year? A framework for an integrated evidence generation strategy. J Comp Eff Res. 2020;9(2):79–82. 31774337 10.2217/cer-2019-0162

[12] Califf R, Robb M, Bindman A, et al. Transforming evidence generation to support health and health care decisions. N Engl J Med. 2016;375:2395–400. 27974039 10.1056/NEJMsb1610128

[13] Sherman R, Davies K, Robb M, et al. Accelerating development of scientific evidence for medical products within the existing regulatory framework. Nat Rev Drug Discovery. 2017;16:297–8. 28232726 10.1038/nrd.2017.25

[14] Olson M. Developing an integrated strategy for evidence generation. J Comp Eff Res. 2017;7(1):5–9. 29053021 10.2217/cer-2017-0073

[15] Malamis P, Howle J. The limited future of real world data? Applied Clinical Trials. 2022. https://www.appliedclinicaltrialsonline.com/view/the-limited-future-of-real-world-data. Accessed Jan 20, 2023.

[16] Malamis P, Howley J. Current and future use of real-world data. Applied Clinical Trials. 2022. https://www.appliedclinicaltrialsonline.com/view/current-and-future-use-of-real-world-data. Accessed Jan 20, 2023.

[17] Zhao L, et al. Generating model integrated evidence for generic drug development and assessment. Clin Pharmacol Ther. 2019;105(2):338–49. 10.1002/cpt.1282. 30414386 10.1002/cpt.1282

[18] Sax FL, et al. Value-based planning & drug development productivity. Appl Clin Trials. 2016;25(4/5):26–34.

[19] Rath B, Surjit KK. Real-world data analytics in global pharmaceutical marketing. ICFAI Univ J Knowl Manage. 2016;14(2):48–59.

[20] Kish J, et al. Use of real-world evidence in clinical decision making by community oncologists. Value Health. 2018;21. 10.1016/j.jval.2018.04.276. S48-S48.

[21] Gill JL, et al. Real world evidence in Europe - The results of an expert survey. Value Health. 2017;20(9):A655. 10.1016/j.jval.2017.08.1554.

[22] Kish JK, et al. PCN526 physician acceptance of real-world evidence in drug approval and labeling. Value Health. 2019;22:S540–540. 10.1016/j.jval.2019.09.717.

[23] Amin P, Nam S, Perez L, Smith J. Integrated evidence generation: A paradigm shift in biopharma., McKinsey, Company. December 2, 2021. https://www.mckinsey.com/industries/life-sciences/our-insights/integrated-evidence-generation-a-paradigm-shift-in-biopharma. Accessed March 30, 2023.

[24] Levitan B, Getz K, Eisenstein E, Goldberg M, Harker M, Hesterlee S, Patrick-Lake B, Roberts JN, DiMasi J. Assessing the financial value of patient engagement: a quantitative approach from CTTI’s patient groups and clinical trials project. TIRS. 2018;52(2):220–29. 29714515 10.1177/2168479017716715 PMC5933599

[25] DiMasi JA, Wilkinson M. The financial benefits of faster development times: integrated formulation development, real-time manufacturing, and clinical testing. TIRS. 2020;54:1453–60. 32500448 10.1007/s43441-020-00172-w

[26] DiMasi JA, Smith Z, Getz KA. Assessing the financial benefits of faster development times: the case of single-source versus multi-vendor outsourced biopharmaceutical manufacturing. Clin Ther. 2018;40(6):963–72. 29755005 10.1016/j.clinthera.2018.04.011

[27] DiMasi JA, Smith Z, Oakley-Girvan I, Mackinnon A, Costello M, Tenaerts P, Getz KA. Assessing the financial value of decentralized clinical trials. TIRS. 2023;57(2):209–19. 10.1007/s43441-022-00454-5. 36104654 10.1007/s43441-022-00454-5 PMC9473466

