# Supplementary material for: Assessing the Value of Integrated Evidence Approaches in Drug Development
Source: Ther Innov Regul Sci. 2025 Apr 23;59(4):808–16. doi: 10.1007/s43441-025-00778-y (PMC12181097; doi:10.1007/s43441-025-00778-y)
Supplement: Supplementary file 1 — Supplementary Material 1 [file 43441_2025_778_MOESM1_ESM.pdf]

SUPPLEMENTAL DATA FILE

ASSESSING THE VALUE OF INTEGRATED EVIDENCE APPROACHES IN DRUG DEVELOPMENT

Joseph A. DiMasi,[1*] Melvin (Skip) Olson,[2*] Zachary Smith,[1] Kenneth A. Getz,[1] Gorana Capkun[3*]

[1] Tufts Center for the Study of Drug Development, Tufts University
[2] Olson Strategies GmbH
[3] Merck Healthcare KGaA

*These authors contributed equally to this work

Additional assumptions for eNPV calculations used in the two case studies

Typical development costs for a PK/bioequivalence study and observational of around $3 million were assumed as well as approximately $20 million for a phase II trial. As there might be a long follow-up period for the phase II trial, we assume that 80% of the costs are incurred in the first three years but it should be noted that the results are not very sensitive to different assumptions about how the costs are distributed over the trial duration.

The typical cost of an appropriate phase IIIb trial is estimated to be $8 million in nominal dollars. The assumed duration of the study is two years. The study is assumed to start at the time that the original application for marketing approval is submitted. The original regulatory review is assumed to take 10 months. The assumed probability that the results of the phase IIIb study will be submitted for regulatory approval is 78%, and the assumed likelihood of regulatory approval, if an application is submitted, is 90%. The eNPV is reduced by the cost of the phase IIIb trial discounted back to the start of the trial.

Based on internal data and published company financials, we assume for the base cases that the contribution margin ratio is 43% (difference between revenue and variable cost divided by revenue), the discrete cost of capital is 6.5%, and the effective corporate income tax rate is 18.2%.

The projected costs and returns for both cases are assumed to be given in nominal dollars and are inflation-adjusted for the analyses using the GDP Implicit Price Deflator as a price index. The analyses are conducted with all costs and returns expressed in year 2020 dollars. Forecasted returns in all cases are specified through 2030. Projecting future returns far into the future is fraught with difficulties. However, given that returns can be expected to extend beyond the estimates given here, the results on the increments in value from employing the IEPs should be viewed as conservative. The internal company cost of capital is a discrete (annual) value. Here, the eNPV calculations are done on a continuous basis, so the assumed discrete company cost of capital is converted to an equivalent continuous cost of capital. All costs and returns are discounted back to the start of the implementation of the IEP. The parameter values used for the sensitivity analyses were chosen based on the authors' judgment on what are

reasonably wide ranges for each parameter. The results illustrate the direction and extent of the effects on net financial benefits as assumed parameter values are increased or decreased.

Table S1. Sensitivity Analysis for an Integrated Evidence Plan (all regions) for Lifecycle Management (new rare disease indication) of a Drug Asset (millions 2020 USD)

	eNPV delta	ROI
Base Case	**$78.3 MM**	**30.7x**
Discount Rate		
**5%**	**$70.8 MM**	**27.6x**
**8%**	**$84.6 MM**	**33.4x**
**10%**	**$91.5 MM**	**36.4x**
Contribution Margin		
**33%**	**$59.5 MM**	**23.3x**
**53%**	**$97.1 MM**	**38.1x**
Increase in relative probability of approval		
**5%**	**$72.1 MM**	**28.3x**
**20%**	**$90.5 MM**	**35.5x**

ROI = (eNPV delta)/(eNPV of cost of chart review)
Sales for all markets used
Phase II study conducted for both the base case and IE scenarios

Table S2. Sensitivity Analysis for an Integrated Evidence Plan for Lifecycle Management (expanded patient population) of a Biologic Asset (millions 2020 USD)

	eNPV delta	ROI
Base Case	**$66.6 MM**	**10.8x**
Discount Rate		
**5%**	**$69.4 MM**	**11.1x**
**8%**	**$64.0 MM**	**10.5x**
**10%**	**$60.7 MM**	**10.2x**
Contribution Margin		
**33%**	**$49.7 MM**	**8.1x**
**53%**	**$83.5 MM**	**13.6x**
Cost of Phase IIIb study		
**$20 MM**	**$57.4 MM**	**3.7x**
**$50 MM**	**$34.3 MM**	**0.9x**
**$100 MM**	**-$4.1 MM**	**-0.1x**

ROI = (eNPV delta)/(eNPV of Phase IIIb study cost)
Phase IIIb study conducted for the IE scenarios

Table S3. Sensitivity Analysis for an Integrated Evidence Plan for Lifecycle Management (expanded patient population) of a Biologic Asset (millions 2020 USD) Completed Two Years Earlier

	eNPV delta	ROI
Base Case	**$127.8 MM**	**18.3x**
Discount Rate		
**5%**	**$131.1 MM**	**19.1x**
**8%**	**$124.6 MM**	**17.6x**
**10%**	**$120.6 MM**	**16.7x**
Contribution Margin		
**33%**	**$96.4 MM**	**13.8x**
**53%**	**$159.1 MM**	**22.8x**
Cost of Phase IIIb study		
**$20 MM**	**$117.3 MM**	**6.7x**
**$50 MM**	**$91.2 MM**	**2.1x**
**$100 MM**	**$47.6 MM**	**0.5x**

ROI = (eNPV delta)/(eNPV of Phase IIIb study cost)
Phase IIIb study conducted for the IE scenarios

Costs and returns are first discounted back to the start of Phase IIIb and then capitalized forward to the regulatory submission date for comparative purposes
